# Notes and News

**Published:** 1890-06-28

**Authors:** 


### Notes and News.

Collected and Communicated.

The Bromley and Beckenham Joint Hospital Board hope to shortly erect a steam disinfecting apparatus. There are six patients in the hospital just now, all convalescent; perhaps this is why the nursing staff have been disagreeing, and Mrs. Burne, the matron, and Miss Cranson, the nurse, have both resigned.

The Invalid Children's Aid Association is steadily increasing in size and power, and is doing much to lighten the heavy burden of pain laid on some of our sickly town children. The receipts last year were £972, and the expenses included £445 for the maintenance of children in homes. The Association works in connexion with the Charity Organisation, and is in touch with most of the children's hospitals.

The thirty-ninth report of St. Mary's Hospital is, as usual, very full and very interesting. Last year was a prosperous one for St. Mary's, and the committee took advantage of an increased income to carry out numerous improvements which had long been waiting for such an occasion. The extraordinary expenditure then for 1889 amounted to £21,599, and patients, nurses, and students have all reason to bless the fact of a prosperous year! Some forty new annual subscribers were also obtained last year, and we cannot but hope that St. Mary's will receive better support in this way, for a steady income is much more useful than a fluctuating one.

The first general meeting of the Hospital Saturday Fund was held on June 14th, and it was announced that the annual streets collection should take place on July 12th. Special efforts are being made to reach the servants of large establishments, and school children of all classes. There has been some slight disagreement about the Morley House Home, which has been settled by the appointment of extra trustees. Since the closing of last year's accounts, viz., January 1st last, up to and including May 31st, 3,103 receipts have been issued, and the sum of £4,196 12s. 2½d. received. This sum consists of donations, £26 5s.; from local committees, £111 16s. 1d.; balance from Surgical Aid Committee, £79 4s. 6d.; workshop collections, £3,979 6s. 7½d.

Cookery competitions are great fun. This is, of course, not the proper way to look at them, you ought to seriously consider the appearance of the different dishes; the tasting, alas! is only for the judges. All this week Westminster Town Hall has been full of neat-handed Phyllises, got up in the most dainty aprons, and busy over the most artistic of dishes. School children were also there, deeply engaged in the mysteries of jam turnovers; and gentlemen of foreign appearance wandered around looking loftily on, and were pointed out in whispers as "the *chef* of the Prince of Wales," or "the *chef* of the Duke of Marlborough." Certainly the Universal Cookery and Food Association is to be congratulated on the useful nature of the interesting competition just held. The profits of these competitions are given to charities.

Drinks for the summer weather are a serious question; eating becomes a trying duty during the dog days, and too many of us are apt to indulge in lemon squash or iced B. and S. instead of in a regular substantial meal. It is to our colonies we have to look now for many new inventions, and our Melbourne correspondent long ago bade us note the progress of "Liquor Carnis," an invention of Dr. Caffyn's, and which is now being manufactured at Deptford. The first use of Liquor Carnis is for invalids, but our Melbourne correspondent bids us try a spoonful of it in our lemonade during the summer months; then the drink will not only assuage thirst but give strength. It is not so very long since Dr. Caffyn came over to England full of enthusiasm, and he ought surely to be satisfied by the reception here accorded to his useful invention.

Her Royal Highness Princess Louise paid a visit to Forest Hill on Tuesday, the 17th inst., the occasion being the laying of the foundation-stone of a new building for the Girls' Home. The Princess, who was accompanied by the Marquis of Lorne, received a right royal welcome, the main streets being most profusely decorated and the line of route was crowded with thousands of spectators. An address was presented to the Princess Louise by Lord Lewisham. After laying the stone Her Royal Highness received purses from a number of children and from representatives of various Sunday-schools in the neighbourhood, and also from local tradesmen. It is intended that the new building shall accommodate thirty girls, and the cost will be £4,000. The total amount received or promised up to the close of the ceremony was nearly £2,200. The Boys' Home is a separate building some little distance away, and has forty inmates.

Messrs. Mawson, Swan, and Morgan, of Newcastle, are going to publish lithographed facsimiles of a manuscript volume about three centuries old, called "Ye Apothecarie." It is full of the most extraordinary prescriptions, and will do much to puff up the pride of the modern man of science. Only it is probable that our pharmacopœia of to-day will be held in derision some 200 years hence. Here is a specimen of "Ye Apothecarie" of the reign of Queen Elizabeth: "To know whether a sick man shall liue or die certenly prooued manie tymes.—Take a penny weight of land cressede and giue ye sick to eate three daies togeather, fasting, and to drinke a drafte of water after it or wine if he cast it up he shall die, or els take tormentell bayberries and mirre and ℥j make these into fine powder, mix them well togeather geue ye sick of it to drinck in stale ale ℥j at a tyme of he cast it upp he dieth of the same sicknes, if he reteme yt he shall liue; the bayes purge, the turmentall voideth all venome and rawe meates lying in the stomak, and ye mirre suffreth no corruption in the body of man." Some of us would rather remain ignorant of the issues of life and death than have our apothecaries experiment on us with herbs and stale ale.

The following vacancies are announced this week, the amount of the salary in each case being given after the name of the institution :—

**Resident Medical Officers.**

Suffolk General Hospital, Dispenser—£40, board, &c.

Hospital for Women, Soho Square, House Physician—£30, board, etc.

London Throat Hospital, House Surgeon.

Monsall Fever Hospital, Medical Officer—£250, board, etc.

Homerton Fever Hospital, Clinical Assistants—board, etc.

Bootle Borough Hospital, Assistant House Surgeon—£40, board, etc.

Wellingboro' Medical Institute, Medical Officer—£280, rooms, etc.

**Non-Resident Medical Officers.**

Lincoln Odd Fellows, Junior Medical Officer—£120.

Gordon Hospital, Assistant Surgeon.

The Zenana Bible and Medical Mission issues a bulky report, which, by maps and illustrations, does its best to interest the public. The foundation-stone of a new building for a hospital was laid at Benares last year; and arrangements are well forward for the Lady Kinnaird Memorial Hospital at Lucknow. The society contemplates building a hospital at Patna, also one in North Ceylon; £1,000 is in hand for the former, and £2,000 is promised towards the latter work. Special efforts are being made to increase the income of the society to £20,000, which will enable the committee to place twenty more English lady missionaries in the field. The society has a staff of sixty-one European missionaries, and there seems to be plenty more women ready to go through the necessary training, and exile themselves in India in order to spread the work. The medical staff consists of five ladies, four of whom are L.R.C.P. and L.R.C.S. of Edinburgh, and one is M.B. and B.S. of London.

The annual report of Walsall College Hospital is always interesting, and specially so this year, owing to the new arrangement by which three acting and three deputy surgeons were appointed on March 3rd, 1889. Walsall Hospital has ever been famed for its surgical success, and under the new régime this prestige seems likely to continue. From October 12th, 1863, to December 31st, 1889, there have been 124 amputations—namely, 79 of legs, and 45 of arms; and of these 124 cases, only 17 terminated fatally, and only one of the fatal cases (or 1 in 124) died of pyæmia. In two of the cases that terminated fatally, the patients died of tetanus; and in every case that terminated fatally there were other and extensive injuries in addition to those necessitating the amputation; and all the cases except three were railway accidents; two were cases in which the patients had been crushed in coal-pits; and in one case the patient, in addition to having both his limbs crushed by machinery, had his ribs fractured and other internal injuries. The income last year was £1,661, of which £366 was from workmen's collections.

The Felixstowe Convalescent Home has to rather curtail its benefits this year, in order that it may blossom out into greater glory in the future. Last year a munificent offer was made by a gentleman interested in the home to build, at a cost of £2,000, a new wing for women, containing thirty beds, to take the place of the old building, which was in many respects unsuitable for the purposes of a convalescent home. The committee at once accepted this generous offer, and decided to take the opportunity of providing in addition, at the same time, suitable baths and lavatories, and of making other improvements and alterations, so that the whole of the new building may be available for occupation by female patients. The new building is in course of erection, and the men's wing has at present to give up a few of its beds to the women. The alteration should be completed by September. The matron, Mrs. Pryke, has been in the habit of holding an annual sale during the bathing season when the town is full; as this is impossible this year, she is issuing collecting cards for the furnishing of the new wing. Old friends take note.

After an important debate before Congregation at Oxford, on June 17th, it was decided by 75 votes to 58 to promulgate the statute admitting women to medical degrees. In consequence of Professor Case's declared opposition, Congregation House was crowded when the Provost of Queen's, on behalf of the Council, moved the adoption of the statute. He declared himself in favour of women doctors for women, and stated that in his own experience he had known a woman who had died because she objected to be examined by a male practitioner. The Rector of Exeter, and Professor Pelham, and Sir Henry Acland supported the motion, the last-named remarking that, as senior examiner, he could assure Congregation there would be no difficulty in the examination of the women, and mentioned that the late Sir Salar Jung, in conversation with him, had stated that there was urgent need for over a thousand women doctors in India alone. Professor Case replied to the various speeches, and the voting resulted as stated above. This statute will have to come before the larger House of Convocation before finally becoming the statute of the University.

On Tuesday, June 10th, the new Sick Hospital erected at Paisley, at a cost of £8,000, for the accommodation of sick poor in the Abbey Parish, was formally opened. The Hospital, which has been erected to the east of the poorhouse, is a fine and substantial building, well fitted in every way to carry out the objects which the Parochial Board have in view, and is, by the largeness of its accommodation and the capabilities of the trained nurses in charge, well suited to give the best attention and comfort to the inmates, and, where possible, to effect a restoration to health. The main building of the Hospital is three storeys in height, and running to the rear at either side are wings of two storeys. The top flat of the main erection is set apart for the accommodation of the nursing staff, the remainder being devoted to the patients. There are four large wards, besides several small ones, the total number of beds being over a hundred, of which sixty are presently occupied. The public were invited to inspect the building from two o'clock until five, and during that time large numbers of county gentlemen and others visited the place. On every hand an opinion was expressed complimentary to the Abbey Board for their efforts to alleviate the sufferings of the poor. It was stated that a number of the visitors had expressed their intention to send in pictures and ornaments for the adornment of the wards.

